# The function of the inter-alpha-trypsin inhibitors in the development of disease

**DOI:** 10.3389/fmed.2024.1432224

**Published:** 2024-08-01

**Authors:** Xin-feng Zhang, Xiao-li Zhang, Li Guo, Yun-ping Bai, Yan Tian, Hua-you Luo

**Affiliations:** ^1^Department of Gastrointestinal and Hernia Surgery, The First Affiliated Hospital of Kunming Medical University, Kunming, China; ^2^Department of Otolaryngology, The First Affiliated Hospital of Kunming Medical University, Kunming, China

**Keywords:** inter-α-trypsin inhibitor (IαI) family, bikunin (BK), ITIHs, hyaluronic acid (HA), extracellular matrix (ECM)

## Abstract

Through the formation of covalent connections with hyaluronic acid (HA), the inter-α-trypsin inhibitor (IαI) family collaborates to preserve the stability of the extracellular matrix (ECM). The five distinct homologous heavy chains (ITIH) and one type of light chain make up the IαI family. ITIH alone or in combination with bikunin (BK) has been proven to have important impacts in a number of earlier investigations. This implies that BK and ITIH might be crucial to both physiological and pathological processes. The functions of BK and ITIH in various pathophysiological processes are discussed independently in this paper. In the meanwhile, this study offers suggestions for further research on the roles of BK and ITIH in the course of disease and summarizes the plausible mechanisms of the previous studies.

## 1 Introduction

Members of the ancient and distinctive inter-α-trypsin inhibitor (IαI) family have developed throughout hundreds of millions of years of vertebrate history (1). Hepatocytes are the primary source of the 225 kDa IαI family protein complexes, which are found in the blood at high concentrations of 0.15 to 0.5 mg/mL (2). Human IαI family consists of three different polypeptide chains, namely bikunin (BK), heavy chain 1 and heavy chain 2 (3). IαI family, which makes up the majority of family members in human serum, is thought to be dormant until it enters the target tissue, where it is cleaved by TNF-stimulated gene 6 protein (TSG-6). After that, heavy chains (HCs) are transferred to hyaluronic acid (HA), a significant part of the extracellular matrix (ECM), through the formation of temporary covalent bonds with TSG-6 (4). TSG-6 is essential for the interaction with HA because it facilitates two following ester exchange reactions: it binds HC1 or HC2 of IαI family covalently and then moves them to the HA fraction in this complex, where the heavy chain conjugates and releases free TSG-6 (5, 6). HCs have been found to function as structural proteins that can directly cross-link HA that is secreted (7). IαI HC forms a strong bond with HA produced by fibroblasts in culture (7). In addition, the physiological correlation of HA with members of the IαI HC family has been linked to various cell types that show HA-containing outer membranes. For example, the maturation process of oocytes and experiments *in vitro* culture of mesothial cells (8). In summary, the IαI family of proteins interacts with HA to maintain the stability of the ECM, which is a critical function in numerous illnesses. This work aims to provide an overview of the mechanisms underlying the occurrence and development of IαI family proteins that have been linked to current disorders. Additionally, it offers suggestions for future research on IαI family proteins.

## 2 The structure and function of inter-α-trypsin inhibitor family members

### 2.1 Bikunin

**Figure 1:**
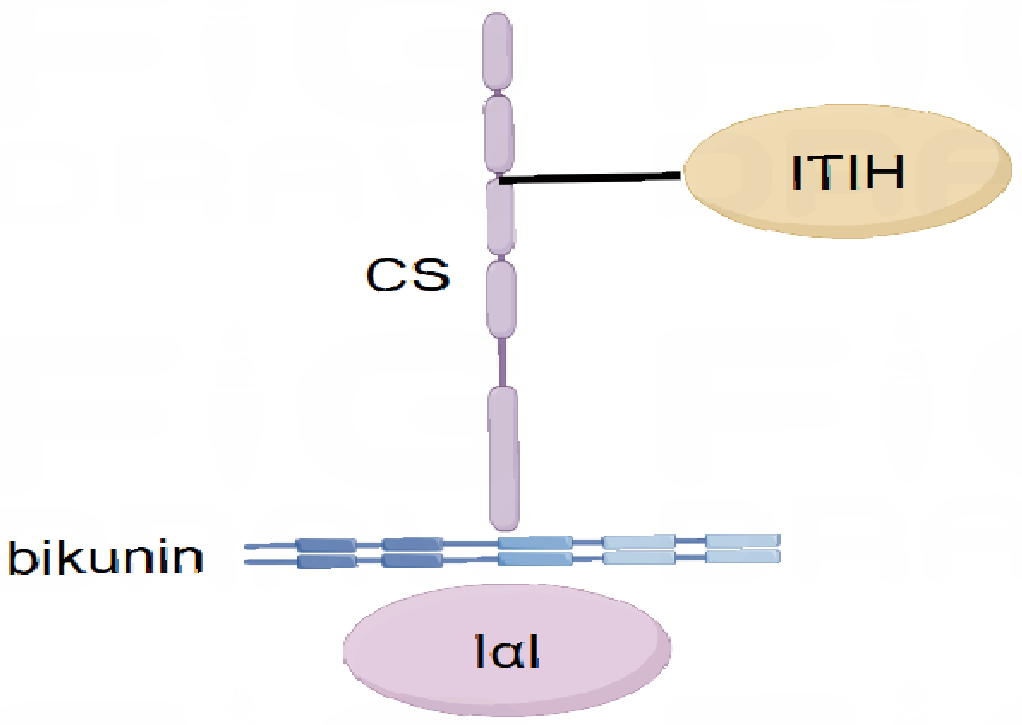
Composition of inter-a-trypsin inhibitors and serum-derived hyaluronan-associated proteins. The IαI family is assembled from light chain-bikunin and five homologous heavy chains.

Bikunin (BK), the light chain, and five homologous heavy chains come together to form the IαI family (9) (Figure 1). There are various homologous heavy chains (ITIH), and the one that has been discovered to date has five members (ITIH1, ITIH2, ITIH3, ITIH4, and ITIH5), despite the fact that there is only one type of light chain. The α-1-microglobulin/BK precursor (AMBP) encodes the light chain and α-1 microglobulin, a member of the lipid transport protein superfamily independent of the ITI family either physically or functionally (10). The ITI light chain is referred to as “BK” because it has two tandem repeats of a Kunitz-type structural domain (11). Serum proteoglycans generated from liver are known as BK isoforms. They contain chondroitin sulfate (CS) chains, which are mainly esterified by one or two glycoproteins referred to as “heavy chains” (HC). There are three primary serum isoforms that have been extensively documented: pro-alpha-proteinase inhibitor and inter-alpha-trypsin inhibitor, which carry one and two HC, respectively, and urotrypsin inhibitor, which corresponds to BK and is connected to the CS chain (10). These complexes create the characteristic ITI protein-glycosaminoglycan-protein structure (12).

### 2.2 ITIH

Apart from BK, the ITI heavy chain also serves biological purposes (13). Numerous diseases, including inflammatory reactions in local tissues, acute inflammation, tumor growth, and problems connected to psychiatry, have been linked to ITIHs, according to studies (13, 14). Subsequent investigations have also revealed that ITIHs, a main constituent of ITI, plays an important role, either by itself or in conjunction with BK (13). The von Willebrand A-type structural domains and the vault are two of the modules that make up the H chain, which can communicate with the ECM. The carboxyl group of the matching H-chain’s C-terminal aspartic acid residue and the C-6 hydroxyl group of the internal N-acetylamino galactose residue of Bk’s chondroitin sulfate component are what link the H-chain to the protein. ITIHs can undergo an ester exchange reaction to covalently conjugate to locally generated HA. Serum-derived hyaluronan-associated protein is made up of D-glucuronic acid and N-acyl-D-glucosamine, which together make up the desialylated polymer known as HA (2). ITIHs bind covalently to HA to stabilize the ECM (15). Many extracellular proteins released by cells make up the ECM, which controls intercellular communication as well as biological activities (16). Many interacting proteins and proteoglycans that control cellular activity have an impact on the stiffness or rigidity of HA (17, 18). One important element of the ECM is HA (19). Together, these molecules of the ECM—HA, proliferating cells, migration, and tumor metastasis—maintain the stability of the ECM by forming a reticular structure (20, 21). A common ECM component, HA is a high molecular weight polymer that does not need to be modified further (21). Numerous biological processes, including wound healing, differentiation, and cell motility, are facilitated by HA-binding proteins, especially membrane-bound receptors like CD44 and RHAMM (22, 23). For instance, during the healing of skin injury, synthesis is elevated. The covalent transfer of heavy chains from IαI to HA is catalyzed by TsG-6, and the resulting HC-HA complex is engaged in remodeling and inflammatory processes in both healthy and pathological contexts (24). Furthermore, HA participates in angiogenic processes. The size of HA affects its angiogenic potential: short segments of HA produced in inflammation and tissue damage are strongly angiogenic (25), while high molecular weight HA possesses vasopressor qualities (26). After degradation, HA becomes a pro-angiogenic ligand (15). For instance, it has been discovered in earlier research that HA stimulates angiogenesis in lung damage and is even linked to abnormal angiogenesis (17). Increased ITIHs protein may prevent the HA-CD44 and ECM pathways from degrading, which would have anti-angiogenic effects. This could be one of the key ways that the protein ITIHs functions as a putative oncogene.

## 3 Advances in the study of BK and disease

In both physiological and pathological processes, BK has pleiotropic functions. BK appears to be involved in numerous activities, according on genetic studies of mice deficient the protein. Genes related to stress, apoptosis, proteases, aging, cytokines, HA metabolism, and female ovulation processes are dysregulated when BK is absent (27, 28). Subsequent research revealed that female mice deficient the BK gene exhibit considerably lower fertility (28). This results from a malfunction in the lateral protein precursors’ ability to form compounds with hyaluronic acid in the ovarian mound before ovulation. Therefore, hyaluronan, which is necessary for mammalian ovulation and fertilization, requires the delivery of serum-derived hyaluronan-related proteins by the chondroitin sulfate part of BK (29). Concurrently, research has produced intriguing findings on the potential of the medication BK to lower the risk of preterm labor and enhance neonatal outcomes (30).

Furthermore, in pancreatitis, septic shock, and rheumatoid arthritis, it has been demonstrated that the BK core protein inhibits inflammation-associated proteases such trypsin, elastase, and fibrinolytic enzymes (31, 32). As an anti-inflammatory, BK prevents the production of cytokines that are triggered by lipopolysaccharide (LPS). Through extracellular signal-regulated kinase signaling (ERK), calcium endocytosis, and endotoxin receptors, BK suppresses the generation of cytokines. Endotoxin stimulates calcium inward flow, generates phosphorylated ERK, and activates multiple transcription factors, including nuclear factor kappa B and early growth response-1, through endotoxin receptor signaling, all of which support the development of cytokines (33). It was discovered to prevent the generation of pro-inflammatory cytokines in a number of cell types in cellular tests (3, 34). Mice injected BK showed a decrease in multiple inflammatory markers in animal models of inflammatory disorders (35). Furthermore, BK knockout mice, Bik (−/−), were used for *in vitro* cytokine experiments and *in vivo* animal models. It was discovered that Bik (−/−) mice induced higher levels of endotoxin-induced death when compared to wild-type (Wt) mice; that application of BK significantly reduced LPS-induced lethality; and that endotoxin significantly increased endotoxin-induced lethality when compared to Wt mice. BK combined with application of BK inhibited the levels of these cytokines; additionally, BK inhibited endotoxin-induced up-regulation of cytokine expression by suppressing macrophage phosphorylation of ERK1/2, JNK, and p38; this implies that BK has a major anti-inflammatory function (36). In different acute and chronic inflammatory reactions, BK is a non-invasive circulating or urine biomarker (37).

BK has been found in cancer studies to inhibit tumor cell invasion through direct inhibition of fibrinolytic activity associated with tumor cells as well as urokinase-type fibrinogen activator (UPA) expression at the gene and protein levels, potentially through inhibition of MAP kinase signaling cascades and/or CD44 dimer. By interacting with different cell types’ cartilage junction proteins and BK receptors, BK can be suppressed in order to prevent cell invasion (38). Furthermore, ovarian cancer cells’ gene expression patterns are changed by BK, which prevents tumor cell invasion (39). In animal investigations, it was discovered that the exogenous injection of BK inhibited the growth of intraperitoneal ovarian tumors as well as peritoneal disseminated metastases (40). Treatment with BK in the adjuvant setting and/or in conjunction with cytotoxic medicines to enhance therapeutic efficacy may be helpful in postponing the beginning of metastases in patients with advanced ovarian cancer (41). Patients with ovarian cancer who had preoperative BK concentrations higher than 11.5 μg/mL were shown to have a significantly better prognosis than those with lower amounts. Patients with pretreatment values of 11.5 μg/mL had 2.2-fold higher Hazard Ratios (risk of mortality) than those with concentrations of more than 11.5 μg/mL of BK. Patients in both groups had a median survival of 26 months and more than 60 months, respectively (42). This implies that BK has a critical function and importance in determining the prognosis of ovarian cancer patients. Remarkably, there has been no report of BK overexpression being linked to human pathology (1).

**Figure 2:**
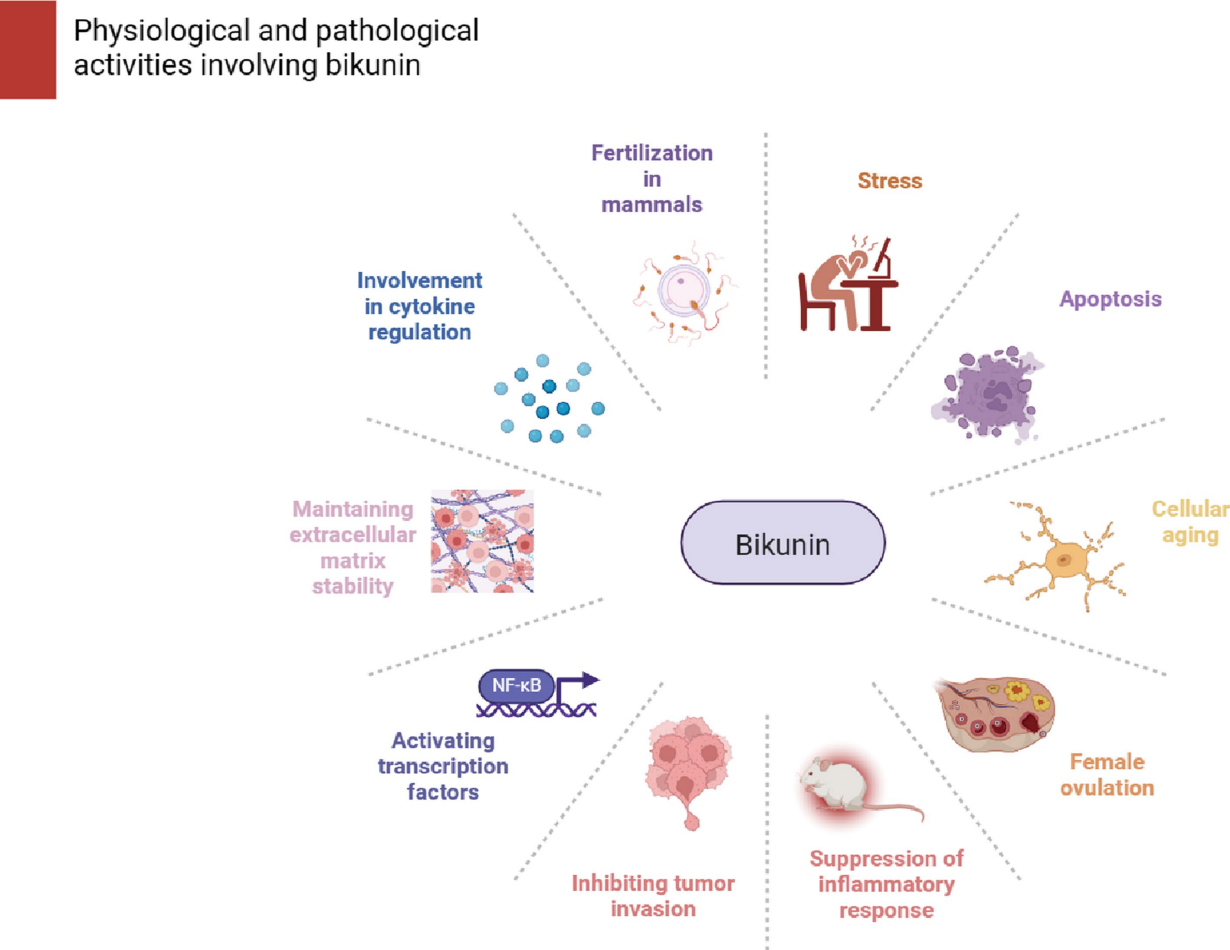
Stress, apoptosis, proteases, aging, signaling molecules, cytokines, HA metabolism, and female ovulation processes are all impacted by bakunin. Its responsibilities and functions as an anti-inflammatory and anti-tumor invasion protein are also significant.

The above cellular and animal experiments suggest that BK has potential medical value. It also plays a significant role in mammalian ovulation and even directly affects fertility in knockout mice. Additionally, BK has important roles and functions as an anti-inflammatory protein and an anti-tumor invasive protein. These findings may lead to new ideas for diagnostic and therapeutic approaches in the future gene therapy of ovarian cancer, inflammation, and infertility. It might offer suggestions for later gene therapy treatments for ovarian cancer, inflammation, and infertility. Figure 2 presents a summary of the physiological and pathological processes that are mediated by BK.

## 4 Developments in the understanding of ITIH proteins and illnesses

ITIH has lately been found and thoroughly explained to be involved in several pathophysiological processes, such as inflammation and carcinogenesis (9, 43). Depending on the circumstances, these proteins may be positively or negatively regulated; nonetheless, there is compelling evidence that every member of the ITIH family is crucial to the development of tumors and cellular malignant processes (9, 44). ITIH proteins function as both pre- and anti-inflammatory acute phase proteins during inflammation, which leads to contradictory functions in the process. While the H3 chain is up-regulated and the related molecules behave as positive acute phase proteins, the H2 and BK chains are down-regulated and the associated molecules behave as negative acute phase proteins in acute inflammation, Inflammatory situations do not seem to have an impact on H1 chain (45). Strong evidence suggests that genes in the ITIH family may be tumor suppressors because these genes are highly down-regulated in a range of human solid tumors, including lung cancer, breast cancer and colon cancer (9). ITIH proteins stabilize the ECM in a way that inhibits tumor growth, which plays a significant role in carcinogenesis (46). ITIH’s covalent binding to HA and its capacity to maintain the ECM are two potential pathways. ITIH2 expression and estrogen receptor expression are substantially associated (*p* = 0.001) in breast cancer, according to a number of prior research. Cancer invasion and motility have been shown to be inhibited by estrogen (47). Because ITIH2 has an estrogen-binding structural domain that profoundly affects ECM integrity and may therefore be crucial for tumor growth and metastasis, there used to be a high correlation between ITIH2 expression levels and estrogen levels (48). ITIH2 is expressed in low-grade CNS cancers and normal brain tissues; however, it is not expressed in glioblastomas, especially glioblastoma multiforme, which is a highly invasive CNS tumor. This suggests that ITIH2 may have an anti-invasive function (49). The ITIH family gene has been linked in certain studies to shared genetic risk factors for schizophrenia and depression, in addition to its significant function in inflammation and malignancies. These findings imply that the ITIH family may potentially be implicated in the pathophysiology of psychiatric disorders (50). Furthermore, aberrant expression of ITIH family proteins has been found in neurodegenerative diseases (51, 52). The following table provides a summary of the roles and possible mechanisms of action of heavy chain participation (see Tables 1–5).

**Table 1:** Functions and potential mechanisms of ITIH1.

	Functions	Mechanisms	References
ITIH1	Insulin sensitivity	ITIH1 binding directly to HA on skeletal muscle and adipose tissue surface causes ECM stability and subsequent insulin resistance Increased ITIH1 may impact β-cell activity	(16)
ITIH1	Indicators for ankylosing spondylitis diagnosis	Markedly elevated in ankylosing spondylitis patients, *p* = 0.035 ROC distinguished between ankylosing spondylitis patients (AUC = 0.98)	(53)
ITIH1	Evaluation of hepatic fibrosis	Liver fibrosis development and impaired ECM stability are linked to lower ITIH1 levels	(54)
ITIH1	Control of adherence of leukocytes	Adhesion of the inflammatory HA is regulated by thrombin cleavage of ITIH1	(55)
ITIH1	Modulator of immunity	Blocks the C3 complement pathway	(56)
ITIH1	Potential biomarker for hepatocellular carcinoma that is both diagnostic and predictive	Hepatocellular carcinoma patients have much lower levels of ITIH1 expression, and this downregulation is detrimental to the prognosis of these patients	(57)
ITIH1	Involved in the development of hepatocellular carcinoma	By stimulating the PI3K/AKT signaling pathway and epigenetically suppressing ITIH1 transcription, KDM5C can encourage the malignant progression of hepatocellular carcinoma	(58)
ITIH1	Markers for radiographic knee osteoarthritis development prediction	Shows promise for enhancing clinical practice radiographic knee osteoarthritis incidence prediction	(59)
ITIH1	Potential biomarkers for ovarian cancer	Variations in expression in the serum of patients with ovarian cancer	(60)
ITIH1	Biomarker for coronary heart disease risk stratification	When paired with additional proteins, ITIH1 exhibits a Lasso-logistic score that is highly effective in categorization (cross-validated area under the curve = 0.74)	(61)

**Table 2:** Functions and potential mechanisms of ITIH2.

	Functions	Mechanisms	References
ITIH2	Increases intercellular adhesion, suppresses cell proliferation, and prevents glioblastoma invasion	Reduces the activity of the PI3K/AKT signaling pathway	(49)
ITIH2	Biomarker for multiple sclerosis in children	Patients with pediatric multiple sclerosis had considerably reduced serum levels of ITIH2 protein	(62)
ITIH2	Pancreatic cancer diagnostic biomarker	Area under the curve = 0.947; ROC separates pancreatic cancer from chronic pancreatitis	(63)
ITIH2	Potential candidates associated with diabetic retinopathy	Comparing the protein profiles of vitreous humor in patients with idiopathic macular lentigines who are not diabetics, it was discovered that they had significantly decreased expression of ITIH2 protein	(64)
ITIH2	Indicators of serum proteins for osteoarticular. Tuberculosis detection	For the diagnosis of osteoarticular tuberculosis, the AUC of ITIH2 was 0.7167 (95% CI: 0.5846–0.8487)	(65)
ITIH2	Hinders the growth of mother cells in gliomas	Tumor cell contact is inhibited by gonadotropin-releasing hormone interaction	(66)

**Table 3:** Functions and potential mechanisms of ITIH3.

	Functions	Mechanisms	References
ITIH3	Play in the development of the human brain	Differential spatiotemporal expression of ITIH3 in the developing human brain is shown by analysis of spatiotemporal histology data, which indicates that the SNP locus rs25352629 of ITIH3 is a susceptibility variable for autism	(67)
ITIH3	Intrahepatic cholestasis in pregnancy: diagnostic indicators	For the diagnosis of intrahepatic cholestasis in pregnancy, the AUC of ITIH3 was 0.8163	(68)
ITIH3	Strongly correlated with a history of attempted suicide in bipolar illness and schizophrenia cases	After multiple testing was taken into account, there was a substantial correlation found between the risk allele for the SNP locus rs2239547 of ITIH3 and a history of suicide attempts	(14)
ITIH3	Strongly correlated with the likelihood of getting schizophrenia	The ITIH3 SNP locus rs3617 may impact neurodevelopment and protein function, raising the risk of schizophrenia	(69)
ITIH3	Potentially forecast ovarian cancer’s susceptibility to cisplatin	Platinum resistance and a poor prognosis are strongly correlated with low expression of ITIH3 protein in ovarian cancer tissues	(70)
ITIH3	Unknown hereditary susceptibility to myocardial infarction	In human atherosclerotic lesions, macrophages and vascular smooth muscle cells express the ITIH3 protein	(71)
ITIH3	It is a helpful biomarker for gastric cancer early detection Assesses the likelihood of gastric cancer	According to ROC, ITIH3 has a maximum sensitivity of 96% and a maximum specificity of 66% in the identification of gastric cancer. Furthermore, patients with early gastric cancer had considerably greater plasma levels of ITIH3 than non-cancerous patients (*p* < 0.001)	(72, 73)
ITIH3	Might not be adequate as a potent biomarker for early gastric cancer detection	Between early and advanced gastric cancer, there was no difference in expression; the AUC value was 0.65 (95% CI: 0.55–0.75)	(74)
ITIH3	Enhances acute and chronic heart failure and hypertrophic cardiomyopathy. Treatment with biomarker candidate	Proteomic analysis was used to identify putative core biomarkers	(75)
ITIH3	A biomarker for the detection of pancreatic cancer	In comparison to controls, expression in pancreatic cancer was 1.80 times greater	(76)
ITIH3	It is a biomarker for the early detection of rheumatoid arthritis	Utilizing innovative diagnostic techniques, rheumatoid arthritis diagnosis approaches 100% sensitivity and specificity	(77)
ITIH3	Contributes to the development of synovial joint disease	Significant elevation of ITIH3 in synovial joint diseases	(78)

**Table 4:** Functions and potential mechanisms of ITIH4.

	Functions	Mechanisms	References
ITIH4	Pro-inflammatory reaction	Activation of the PGK1-ITIH4 axis causes a pro-inflammatory response	(79)
ITIH4	Cholestatic liver disease biomarkers	Cholestatic liver dysfunction was associated with considerably higher plasma ITIH4 values	(80)
ITIH4	Biomarkers that show how NAFLD progresses and how hepatocellular carcinoma develops as a result	Compared to patients with simple steatosis and virus-associated hepatocellular carcinoma, patients with hepatocellular carcinoma-NAFLD had considerably higher serum ITIH4 levels Following hepatectomy, patients with hepatocellular carcinoma-NAFLD who had greater serum ITIH4 levels had a worse prognosis	(81)
ITIH4	A possible therapeutic target to prevent the spread of tumors	HuH7 cell movement was markedly reduced by ITIH4 overexpression and increased by ITIH4 knockdown, respectively. Patients with hepatocellular carcinoma who had a good prognosis had tumor tissues with higher levels of ITIH4 expression than those who had a bad prognosis	(82)
ITIH4	Distinguishes multisystemic and unisystemic histiocytosis of Langerhans cells	Significant variations in ITIH4 expression were found between the two illness groups by peptideomics analysis	(83)
ITIH4	Participated in repeated abortions	The activation of the IL-6 signaling pathway by ITIH4^(ΔN688)^ promotes inflammatory responses The long isoforms of ITIH4 have different functions in controlling cell invasion, migration, proliferation, and inflammatory responses	(84)
ITIH4	Beneficial for anticipating and monitoring sepsis	The expression of ITIH4 varies with the stage of sepsis	(85)
ITIH4	Possible prognostic biomarker for psoriasis patients’ liver fibrosis brought on by methotrexate	Patients with psoriasis who had liver fibrosis brought on by methotrexate had abnormally high levels of it in their urine	(86)
ITIH4	Early gastric cancer biomarkers	When compared to patients receiving will-care controls, patients with early gastric cancer had noticeably higher serum levels of ITIH ITIH4 expression increased during *Helicobacter pylori* infection	(87, 88)
ITIH4	Biomarkers for ovarian cancer	Patients with ovarian cancer had far lower ITIH4 expression levels in their urine than did controls	(89)
ITIH4	Evaluation of the diagnostic and prognostic importance in individuals with hepatitis B-associated hepatocellular carcinoma and liver cirrhosis caused by the virus	Patients diagnosed with hepatocellular carcinoma had lower serum ITIH4 levels, and those with hepatitis B-associated hepatocellular carcinoma also had shorter survival times	(90)
ITIH4	Associated with autoimmune disease	Prevention of neutrophil recruitment in order to reduce inflammatory autoimmunity	(91)
ITIH4	Prognostic markers for aneurysmal subarachnoid hemorrhage in humans (ASAH)	Prevent inflammation Early on ASAH, a substantial drop in serum ITIH4 concentrations was highly correlated with a poor prognosis and the severity of the illness	(92)
ITIH4	Prostate cancer and prostatic hyperplasia identification	Patients with prostate cancer had considerably higher ITIH4 protein levels	(93)
ITIH4	Ischemic acute stroke biomarkers	As the acute ischemic stroke condition progressed, ITIH4 eventually returned to normal	(94)
ITIH4	Preventing the growth of melanoma	A finding not further explained in the text	(95)
ITIH4	Monitoring of patients with postoperative breast cancer	ITH4 was substantially lower in postoperative breast cancer patients than in controls	(96)
ITIH4	Accurately forecasting hyperlipidemia	C/T polymorphism of single nucleotides at IVS17 + 8 of ITIH4. Ninety percent of those missing the T allele went on to develop hypercholesterolemia. Of those with the T allele, only 10% experienced hypercholesterolemia (*p* < 0.0001)	(97)
ITIH4	Potential Alzheimer’s disease serum biomarkers	Increased intact size of ITIH4 protein and decreased fragmentation of ITIH4; increased ITIH4 expression	(51, 52)
ITIH4	Interstitial cystitis biomarkers	ITIH4 plasma concentrations were greater in the patients (*p* = 0.019)	(98)

**Table 5:** Functions and potential mechanisms of ITIH5.

	Functions	Mechanisms	References
ITIH5	Indicator biomarkers for bile duct cancer diagnostics	In comparison to the control group, which included people with hepatocellular carcinoma, benign illness, chronic hepatitis B, and healthy persons, the cholangiocarcinoma group had greater serum ITIH5 levels. The AUC for ITIH5 varied between 0.839 and 0.851, indicating a significant difference between bile duct cancer and the control group	(99)
ITIH5	Prevention of the development of pancreatic cancer	Prevention of the spread of pancreatic cancer	(100)
ITIH5	Prevention of the development of bladder cancer	Overexpression of ITIH5 in bladder cancer cells prevented colony spreading and cell migration	(101)
ITIH5	Prevention of the spread of breast cancer	ITIH5-overexpressing breast cancer cells nearly totally failed to develop lung metastasis in a mouse metastasis model	(102)
ITIH5	Stops the spread of cervical cancer	Reduced the growth and invasiveness of tumor spheres to a large degree, which increased the rate of death in cervical cancer cells	(103)
ITIH5	Adipokine released by adipocytes	By comparing gene expression in subcutaneous and visceral fat microarray investigations in obese and lean subjects, ITIH5 was discovered as a novel adipokine	(104)
ITIH5	Involved in wound healing	The transformation of fibroblasts into myofibroblasts, which is dependent on growth factor β1, requires the interaction of ITIH5 with cell surface HA	(105)
ITIH5	Involved in breast cancer development	Breast cancers exhibit a marked downregulation of ITIH5 expression. While ITIH5 expression is either consistently missing or significantly downregulated in invasive ductal carcinomas, normal breast epithelial cells express ITIH5 substantially. Both benign breast cell lines and breast cancer cell lines did not exhibit ITIH5 gene expression. Breast cancer development may be linked to the lack of ITIH5 expression	(106)
ITIH5	Prevention of the development of cervical cancer	ITIH5 overexpression resulted in a marked decrease in cell migration and clone formation as well as a considerable inhibition of cervical cancer cell proliferation	(107)
ITIH5	Prevents the growth of colon cancer cell	Colon cancer cells were unable to proliferate when ITIH5 was overexpressed	(108)
ITIH5	Prevention of the development of pancreatic cancer	ITIH5 may obstruct many oncogenic signaling pathways, including as the PI3K/AKT pathway. Changes in cell migration and the creation of local adhesions may result from this. These alterations in the cells could be connected to ITIH5’s ability to prevent metastasis in pancreatic cancer	(109)
ITIH5	Prevention of the development of non-small cell lung cancer	Lower expression of ITIH5 is linked to a worse prognosis in non-small cell lung cancer	(110)
ITIH5	Responsible for the emergence of clinical metabolic abnormalities and obesity	Obese people express ITIH5 more than lean people do, and dieting-induced weight loss reduces ITIH5 expression	(111)
ITIH5	Contribute to the pathophysiology of congenital megacolon	Elevated ITIH5 expression prevents cell migration and proliferation	(112)

The ITIH family has five homologous variants that are encoded by various genes. Through an overview of current research pertaining to the ITIH family, we discovered that ITIH plays a role in numerous pathophysiological mechanisms. These comprise immunological responses, tumor growth, psychological issues, and inflammation. These protein families play a role in the modification of extracellular structures necessary for cell migration and the growth of malignant tumors. ITIH family has intricate functions. Various disease processes may involve distinct heavy chain proteins. Different diseases may involve the same heavy chain protein. This implies that ITIH family may not be as accurate a diagnostic or prognostic marker for some disorders. Large-scale multicenter clinical trials are required in the future to assess if ITIH family may be used as a prognostic or diagnostic biomarker for specific illnesses. Despite the fact that numerous studies have demonstrated that the ITIH family prevents the growth of different types of solid tumors. The ITIH family is a putative oncogene, but further investigation and analysis are required, and it’s still unclear how it works.

## 5 Summary

To jointly preserve the stability of the ECM, members of the IαI family bind covalently to HA. Hence the characteristics play a role in several physiological and pathological processes. The IαI family contains the protein BK, which is important for mammalian ovulation and human cancer. It is also an anti-invasive protein. The ITIH family is important for inflammation, immunity, psychiatric disorders, tumorigenesis, and development. Although BK has been extensively researched, its exact mechanism of action in the pathophysiological processes involving the ITIH family is still unclear. In order to examine the possible mechanisms and offer a theoretical foundation for targeted therapy of the associated disorders, a significant amount of research is required going forward. In addition, existing studies have shown that BK and ITIH family have been found to play a role in both inflammation and tumors, but whether there is an interaction between the two remains unclear.

## Author contributions

X-fZ: Investigation, Writing – original draft, Data curation. X-lZ: Writing – original draft, Data curation, Investigation. LG: Writing – original draft. Y-pB: Writing – original draft. YT: Supervision, Writing – review & editing. H-yL: Supervision, Writing – review & editing.
